# Cardiovascular–kidney–metabolic syndrome through the lens of gut‑derived uremic toxins

**DOI:** 10.1080/19490976.2026.2685906

**Published:** 2026-06-15

**Authors:** Chudan Xu, Matthew Snelson, Francine Z. Marques

**Affiliations:** a Hypertension Research Laboratory, Department of Pharmacology, Biomedicine Discovery Institute, Faculty of Medicine, Nursing and Health Sciences, Monash University, Melbourne, Australia; b Victorian Heart Institute, Monash University, Melbourne, Australia; c Nutrition and Health Innovation Research Institute, School of Medical and Health Sciences, Edith Cowan University, Joondalup, Australia; d Baker Heart and Diabetes Institute, Melbourne, Australia

**Keywords:** Gut microbiome, uremic toxins, cardiovascular-kidney-metabolic syndrome, microbiome-targeted therapy

## Abstract

Cardiovascular–Kidney–Metabolic (CKM) syndrome represents a complex, interconnected cluster of cardiovascular disease, chronic kidney disease, and metabolic disorders such as obesity and type 2 diabetes. These conditions share overlapping metabolic, inflammatory, and vascular pathways, with the gut microbiome increasingly recognised as a key contributor and common underlying risk factor. Uremic toxins, traditionally considered waste products of host and microbial metabolism, are now recognised as active mediators of tissue damage across the CKM spectrum, particularly in the context of impaired renal function. Their production and accumulation are amplified by disrupted intestinal barrier integrity, chronic inflammation, and reduced renal clearance, collectively driving systemic toxicity throughout the CKM continuum. This review explores the origins and impact of gut-derived uremic toxins, including trimethylamine-*N*-oxide (TMAO), indoxyl sulfate (IS), *p*-cresol sulfate (PCS) and its associated metabolites, *p*-cresol and *p*-cresol glucuronide (PCG), phenylacetylglutamine (PAGln), and imidazole propionate (ImP) within the context of CKM syndrome. These toxins originate from an imbalanced gut microbiome, often shaped by poor diets, such as low-fibre and high-meat intake. We discuss their production by the microbiome and their roles from cardiovascular, renal, and metabolic perspectives and highlight emerging microbiome-targeted strategies to mitigate their pathogenic effects.

## Graphical abstract

**Figure:**
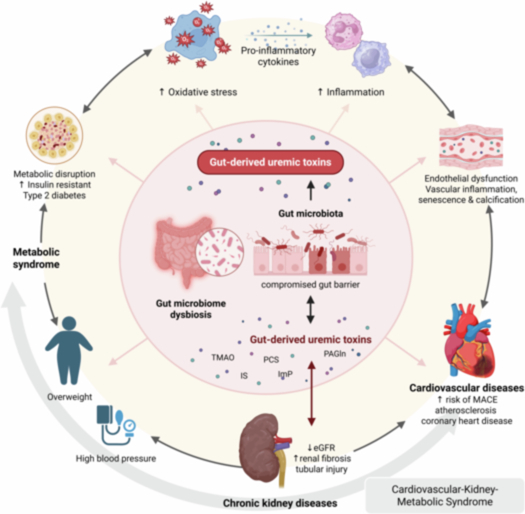

## Introduction

Cardiovascular-kidney-metabolic (CKM) syndrome, a concept introduced by the American Heart Association in 2023,^1^ is the interconnected pathological cluster involving cardiovascular disease (CVD), chronic kidney disease (CKD) and metabolic syndromes, such as obesity and type 2 diabetes (T2D).^2^ These conditions impose some of the highest healthcare expenditures worldwide,^3-6^ underscoring the urgent need to lessen their clinical and economic impact. The CKM syndrome conceptual framework reflects growing recognition that these conditions often co-occur and amplify one another, increasing the risk of cardiac and renal complications as well as premature mortality.^2^ CKM syndrome is driven by overlapping biological pathways, such as chronic inflammation, endothelial dysfunction, and metabolic dysregulation, that create a vicious cycle of damage across organ systems.^2^ Environmental factors, including diet, physical activity, and stress management, influence the risk of developing CKM syndrome and its associated complications.^2^ These factors interact with individual biological predispositions, such as the microbiome, (epi)genetic factors and early life events, which also contribute to the onset and progression of CKM syndrome and its outcomes.^2^ Nonetheless, the dynamic interactions between established risk factors and the biological mechanisms that initiate and propel CKM syndrome remain insufficiently characterized and poorly understood.

The individual aspects of CKM syndrome and its risk factors are closely intertwined through overlapping metabolic, inflammatory, and vascular mechanisms, with the gut microbiome emerging as an additional contributor in the last decade. Growing evidence now identifies the gut microbiome as a major influencer of the risk factors underlying the development of CKM syndrome, such as high blood pressure (BP),^7-10^ and also implicates it in CVD,^11-13^ CKD,^14^ ^,^ ^15^ and metabolic syndrome.^16-19^ One of the primary roles of the gut microbiota is aiding food digestion; for example, they digest complex carbohydrates (e.g., dietary fiber) and dietary proteins that escape digestion in the small intestine, a process made possible by microbial enzymes not found in humans.^20-22^ Through the fermentation of dietary fiber, microbes release metabolites known as short-chain fatty acids (SCFAs), primarily acetate, propionate and butyrate.^23-25^ These metabolites have been recognized for their anti-inflammatory effects in the intestine,^26-28^ ability to lower BP,^23^ ^,^ ^25^ ^,^ ^29^ and modulation of hormonal and lipid/energy homeostasis.^30-32^ Conversely, the gut microbiome can also produce uremic toxin precursors, which are absorbed by the host and converted into uremic toxins including trimethylamine-*N*-oxide (TMAO), indoxyl sulfate (IS), and *p-*cresol sulfate (PCS). These metabolites, derived from microbial metabolism followed by host enzymatic oxidation or conjugation (e.g., sulfation), accumulate as kidney function declines and contribute to a range of biochemical and physiological disturbances.^33-35^ Here, we review and discuss CKM syndrome through the lens of gut microbiome-derived uremic toxins, and explore their emerging potential as therapeutic targets.

### Microbial metabolites and uremic toxins

**Figure 1.**
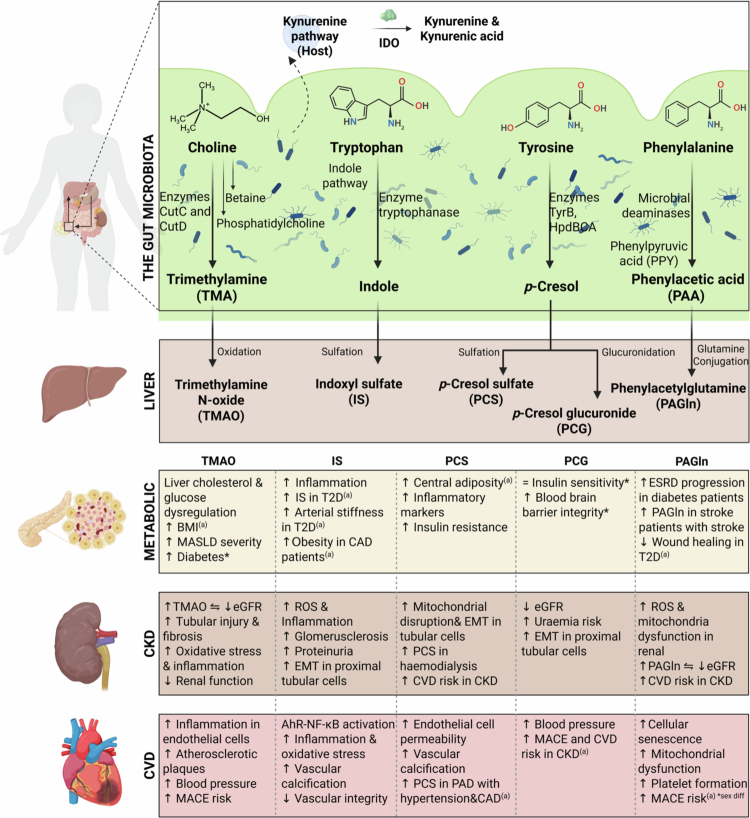
Gut-originated uremic toxins and their roles in cardiovascular-kidney-metabolic (CKM) syndrome progression. The gut microbiome has emerged as a key player in the CKM syndrome. Host dietary patterns and health status shape microbial composition and activity, influencing the generation of uremic toxins, such as TMAO, IS, PCS/PCG and PAGln. These metabolites arise from microbial fermentation of dietary substrates that reach the large intestine and enter the circulation, where impaired renal clearance amplifies their accumulation. The schematic highlights how these toxins act as shared drivers linking gut imbalance to progressive cardiovascular, kidney, and metabolic dysfunction. **Legends:** ↑ increase; ↓ decrease; (a) association-based findings; *controversial or requiring further investigation; *sex diff, potential sex differences. **Abbreviations:** CAD, coronary artery disease; CKD, chronic kidney disease; CVD, cardiovascular disease; eGFR, estimated glomerular filtration rate; EMT, epithelial-to-mesenchymal transition; ESRD, end-stage renal disease; IDO, indoleamine 2,3-dioxygenase; IS, indoxyl sulfate; HpdBCA, *p*-Hydroxyphenylacetate decarboxylase; MACE, major adverse cardiovascular events; MASLD, metabolic dysfunction-associated steatotic liver disease; PAA, phenylacetic acid; PAGln, phenylacetylglutamine; PCG, *p*-Cresol glucuronide; PCS, *p*-Cresol sulfate; ROS, reactive oxygen species; T2D, type 2 diabetes; TyrB, tyrosine aminotransferase; TMA, trimethylamine; TMAO, Trimethylamine *N*-oxide. Figure created in BioRender.

Uremic toxins are organic metabolic waste products that, under normal conditions, are filtered out of the plasma by the kidneys and eliminated through urine. When glomerular filtration or tubular secretion is impaired, these substances cannot be excreted efficiently from the body and, therefore, accumulate in the bloodstream.^36^ Such a condition often elicits inflammation and oxidative stress, affecting other organs, including the kidneys and the cardiovascular system.^36^ Notably, precursors to these uremic toxins are produced as a result of microbial metabolism (Figure 1).

#### What are uremic toxins and their different classes?

Uremic toxins are classified based on their molecular size and protein-binding properties, which affect their removal by dialysis and their excretion. Urea, creatinine, and uric acid are examples of small, water-soluble compounds (size < 500 Daltons) that are easily dialysable toxins.^37^ The middle-sized molecules, such as cytokines and β2-microglobulin, range from 500 to 60,000 Daltons, and some are feasible to be removed with high-flux dialysis. Uremic toxins derived from gut microbial metabolism are predominantly protein-bound compounds that vary in molecular size but bind strongly to albumin, making them particularly poorly cleared by dialysis and, therefore, the most clinically problematic.^37^

#### The emerging role of uremic toxins in CKM syndrome

Uremic toxins are not merely waste by-products, but also active mediators of organ damage in the CKM syndrome, particularly when kidney function is impaired. Their production and accumulation are often amplified by chronic inflammation, altered intestinal barrier function and poor renal clearance, thereby exerting toxic effects across the CKM continuum. For example, uremic toxins such as IS and PCS promote endothelial dysfunction and vascular calcification, increasing the risk of CVD, such as atherosclerosis, arterial stiffness, and heart failure.^38^ Their nephrotoxic effects cause worsened tubular cell injury, renal fibrosis and inflammation, creating a vicious cycle of worsening kidney injury. They also disrupt insulin function, leading to a metabolic imbalance. Therefore, uremic toxins exemplify the integrated nature of CKM syndrome, linking diet, gut microbiota, and organ cross-talk in disease progression. Here, we focus on the uremic toxins TMAO, IS, PCS and its associated metabolites *p*-Cresol and *p*-Cresol glucuronide (PCG), imidazole propionate (ImP), and phenylacetylglutamine (PAGln) within the context of CKM syndrome, discussing their roles from intestinal, cardiovascular, renal, and metabolic perspectives, and outlining potential future strategies for their management in CKM syndrome.

## Trimethylamine *N*-oxide (TMAO)

TMAO, one of the most studied microbially-derived uremic toxins, has emerged as a novel biomarker associated with various human health conditions, particularly those relevant to CKM syndrome. A meta-analysis of 82 individual studies and 18 unique health outcomes reported that plasma TMAO is associated with hypertension, CVD, kidney function, diabetes mellitus and all-cause mortality.^39^ TMAO is a product of the combined metabolic activities of the gut microbiome and the host. Its precursor, trimethylamine (TMA), is a gut-derived metabolite primarily produced by bacterial enzymes CutC and CutD.^40^ TMA is formed from microbial metabolism of choline, phosphatidylcholine, betaine, and _L_-carnitine, which are found in a diet rich in milk, red meat, and eggs.^41^ ^,^ ^42^ The gut microbiota, via CutC and CutD, breaks produces TMA as an intermediate metabolite,^43^ which is then further metabolized by the host hepatic flavin-containing monooxygenase (FMO3) into TMAO.^41^ ^,^ ^44^

*In vitro* screening of 79 isolates from the human intestinal tract identified 6 bacteria that consumed choline and accumulated TMA: *Anaerococcus hydrogenalis, Clostridium asparagiforme, Clostridium hathewayi, Clostridium sporogenes, Escherichia fergusonii, Proteus penneri, Providencia rettgeri,* and *Edwardsiella tarda*.^45^ In a comprehensive multiethnic cohort of 1,653 adults, plasma TMAO concentrations were robustly correlated with the relative abundance of 13 key bacterial genera.^46^ This clinical screening highlighted significant positive associations with prominent TMA-producing and structural taxa, including *Prevotella, Mitsuokella, Fusobacterium*, and *Desulfovibrio*, as well as specific members belonging to the families Ruminococcaceae and Lachnospiraceae (which include many *Clostridium* species).^46^

Another dietary source of TMAO is fish, where it is present as a pre-formed compound rather than generated via microbial metabolism. In marine fish, TMAO functions as an osmolyte and protein stabilizer, with tissue concentrations increasing with the depth at which fish reside, reflecting its role in counteracting hydrostatic pressure.^47^ In human feeding trials, fish consumption produces a rapid rise in circulating TMAO, with plasma concentrations elevated within 15 minutes of intake, consistent with direct intestinal absorption of dietary TMAO, in contrast to the delayed microbiota-dependent production observed following intake of eggs or red meat.^48^ Despite these robust postprandial increases in circulating TMAO, epidemiological evidence consistently demonstrates that habitual fish consumption is associated with reduced cardiovascular and kidney disease risk.^49^ ^,^ ^50^ In the EQUAL study of 737 older adults with advanced CKD, higher TMAO was associated with all-cause mortality, but this association was not observed in patients with concomitantly higher serum 3-carboxy-4-methyl-5-propyl-2-furanpropionate (CMPF), a biomarker of fish intake.^51^ Together, these observations highlight that nutrients act within complex food matrices, where co-occurring bioactive constituents (e.g., n‑3 polyunsaturated fatty acids) may modify biological effects independently of, or in addition to, metabolites such as TMAO.

### TMAO in blood pressure regulation and cardiovascular disease

Mounting evidence consistently suggests that elevated TMAO concentrations and their dietary precursors are associated with increased cardiovascular risk. A meta-analysis of 19 prospective studies, involving 19,256 participants and 3,315 cardiovascular events or deaths, demonstrated that individuals with elevated TMAO levels experienced a 62% increased risk of major adverse cardiovascular events (MACE) and a 63% higher risk of all-cause mortality.^52^ In a dose-response analysis, every 1 μmol/L increase in TMAO was associated with a 2% increase in the relative risk of MACE.^52^ Elevated levels of TMAO precursors, including L-carnitine, choline and betaine, were similarly associated with 1.3- to 1.4-fold increased risk of MACE.^52^ These findings are reinforced by another comprehensive meta-analysis comprising 17 clinical studies (*n* = 26,167), which showed that higher plasma TMAO levels consistently predicted both MACE and all-cause mortality.^53^ Similarly to the other study mentioned above, the prediction was dose-dependent, with mortality risk increasing 7.6% for every 10 µmol/L rise in circulating TMAO.^53^ These associations were further strengthened by an updated meta-analysis showing that elevated TMAO levels were associated with a higher risk of CVD (*n* = 22,945 across 12 studies), an increased risk of CVD mortality (*n* = 11,296 across 8 studies), and raised systolic BP (*n* = 17,369 across 19 studies), while there was no such difference observed in diastolic BP.^39^ Similarly, increased systolic BP was positively associated with higher circulating TMAO levels, alongside greater arterial stiffness, in healthy middle-aged to older adults (45-79 years, *n* = 101) compared with young adults (18-27 years, *n* = 21) in a retrospective observational human study.^54^

In addition, in a prospective study with a three-year follow-up of 2,529 patients with advanced CKD, higher TMAO levels were independently associated with an increased risk of ischemic cardiovascular events after adjusting for all potential cardiovascular risk factors.^55^ Eighty-one patients with stable angina and reduced flow-mediated vasodilation also exhibited higher concentrations of TMAO and the pro-inflammatory cytokine interleukin (IL)-1β, along with significantly lower levels of circulating endothelial progenitor cells.^56^ *In vitro* studies demonstrated that endothelial progenitor cells treated with TMAO increased cellular inflammation and oxidative stress while inhibiting endothelial progenitor cell functions.^56^

In animal studies, TMAO interfered with liver cholesterol metabolism by inhibiting reverse cholesterol transport and bile acid secretion.^57^ In addition, dietary supplementation with choline^57^ or TMAO^58^ independently enhanced the expression of several macrophage scavenger receptors, thereby promoting macrophage cholesterol accumulation, macrophage foam cell formation and atherosclerotic plaques.^57^ ^,^ ^58^ Germ-free mice receiving a fecal microbial transplant from a high TMAO-producing human donor exhibited a markedly enhanced platelet aggregation and increased platelet reactivity, promoting thrombosis formation, compared with germ-free mice receiving fecal microbiota from a low-TMAO-producing human donor.^59^ ^,^ ^60^ Furthermore, oral TMAO (1%) administration aggravated Ang II-induced vasoconstriction and amplified the hypertensive response.^61^ These effects were attenuated by using antibiotic treatment, targeting TMA-producing gut bacteria, showing that TMAO was the key driver of the rise in systolic BP.^61^ Consistently, dietary supplementation with 0.12% TMAO in aging experimental models increased aortic pulse wave velocity and elevated aortic advanced glycation end-products (AGEs), thereby worsening aortic stiffness.^54^ These vascular changes were accompanied by an increase in systolic BP of approximately 10 mmHg in both young and older mice.^54^ Collectively, there is robust evidence that TMAO promotes inflammation and vascular pathophysiology in animal studies and is strongly associated with increased cardiovascular risk in clinical studies.

### TMAO in chronic kidney disease

Unlike IS and PCS, TMAO is not protein-bound and is primarily eliminated by renal tubular secretion or, in end-stage renal disease, by hemodialysis.^62^ TMAO concentrations increase progressively with declining renal function, with levels reported to be up to ~40-fold higher in seven hemodialysis patients prior to dialysis than in six healthy controls.^63^ While initial discussions questioned whether elevated TMAO levels were a cause or consequence of kidney dysfunction, it was suggested that these elevations may reflect impaired renal clearance,^64^ accumulating mechanistic evidence supports a causal role for TMAO in the pathogenesis of CKD. Early studies demonstrated that administration of TMAO or its precursor choline to male C57BL/6J mice reduced renal function, as indicated by elevated cystatin C levels and tubular injury, including increased interstitial collagen deposition and upregulation of kidney injury molecule-1 (KIM-1).^33^ In the male 5/6 nephrectomy rat model, TMAO administration exacerbated renal dysfunction, tubular damage, and increased markers of inflammation and oxidative stress in kidney tissues compared to nephrectomised controls not receiving TMAO.^65^ Similarly, female ApoE knockout mice with adenine-induced kidney injury treated with a gut microbial TMA synthesis inhibitor exhibited reduced renal injury, decreased kidney damage, and significantly attenuated microalbuminuria.^66^ Furthermore, in an ischemia/reperfusion model of acute kidney injury, genetic deletion of FMO3 resulted in lower serum TMAO levels, improved renal function, and reduced tubular injury compared to wild-type male mice.^67^ Collectively, these findings strongly implicate TMAO as a pathogenic factor in both acute and chronic kidney injury, highlighting its potential as a therapeutic target in renal disease.

### TMAO in metabolic syndrome

In a case-control study of 250 adults, higher serum TMAO levels were significantly associated with the risk of metabolic syndrome.^68^ A meta-analysis revealed a positive dose-dependent association between circulating TMAO levels and higher body mass index (BMI).^69^ In a prospective cohort study of 1,964 adults, increased TMAO levels correlated with unfavorable changes in regional fat redistribution, an indicator increasingly recognized as a stronger predictor of metabolic syndrome and CVD risk than BMI or total fat mass.^70^ Notably, these associations were more pronounced in middle-aged and older community-dwelling females (*n* = 1,423), in whom higher serum TMAO was related to increases in both fat-to-lean mass ratio and trunk-to-leg fat ratio, while no significant changes were observed among males (*n* = 541),^70^ highlighting that the role of TMAO might be sex-specific in this context. Furthermore, in a cross-sectional observational study of 137 adults, TMAO levels increased progressively with higher BMI and showed strong positive associations with the visceral adiposity index and the fatty liver index (FLI).^71^ In the same study, researchers also identified sensitive clinical cut-off values for circulating TMAO as an early biomarker to predict metabolic dysfunction-associated steatotic liver disease (MASLD)-FLI (≥8.02 µM) and metabolic syndrome (≥8.74 µM) risk in borderline conditions where overt metabolic syndrome has not yet developed.^71^ Consistently, higher circulating TMAO levels were associated with greater MASLD severity in a larger community-based cross-sectional study (*n* = 1,628).^72^

Moreover, studies have reported higher plasma TMAO levels in patients with diabetes mellitus and its cardiovascular and renal complications; however, findings remain inconsistent, with associations varying by population and adjustment for confounders.^73^ To illustrate, a confined meta-analysis of cohort studies found a significant association between TMAO and diabetes mellitus; this finding was supported by case-control studies but not cross-sectional designs.^39^ Additionally, this significance was lost when evaluated using a dose-response analysis.^39^ Nevertheless, a meta-analysis of 2,180 females reported that higher plasma TMAO concentrations were correlated with an increased risk of having gestational diabetes mellitus.^39^ In T2D patients with advanced CKD (*n* = 20), there was a higher abundance of TMA-producing bacteria, such as *Clostridium* and *Escherichia*, and elevated serum TMAO levels compared with healthy individuals.^74^ Additionally, TMAO showed strong positive correlations with zonulin and lipopolysaccharide (LPS) (markers of gut permeability, inflammation, and endothelial dysfunction),^75^ indicating compromised intestinal barrier integrity in T2D patients with CKD.^74^

Emerging evidence suggests that TMAO can alter glucose metabolism^76^ ^,^ ^77^ and modulate *β*-cell function and insulin secretion.^76^ In a prospective study (*n* = 4,442 older adults), higher plasma concentration of TMAO, carnitine and *γ*-butyrobetaine was each independently associated with elevated fasting insulin levels (insulin resistance marker).^77^ Furthermore, a clinically relevant level of TMAO, similar to that observed in diabetes, reduced glucose-stimulated insulin secretion in both mouse *β*-cell lines and primary islets from mice and humans.^76^ Elevating TMAO levels through a 1% choline-enriched diet in male mice further confirmed these findings, leading to impaired glucose-stimulated insulin secretion and reduced glucose tolerance.^76^ In vitro, mouse pancreatic *β*-cells (MIN6) treated with TMAO disrupted calcium signaling through NLRP3 inflammasome-related cytokines, reduced Serca2 expression, impaired *β*-cell calcium handling, and induced endoplasmic reticulum stress, collectively contributing to *β*-cell dysfunction.^76^ Nevertheless, some argue that the elevated TMAO levels may reflect the hepatic insulin resistance rather than act as a primary driver. Under normal physiological conditions, insulin signaling downregulates FMO3 expression, the hepatic enzyme that converts TMA to TMAO, thus maintaining low TMAO levels.^78^ In dysregulated insulin states, a hallmark of T2D and metabolic syndrome, this regulatory control is impaired, leading to higher FMO3 activity and TMAO levels.^78^ Thereby, elevated TMAO may serve as an indicator of metabolic dysfunction rather than a direct causative factor; however, further evidence is needed to clarify the role of TMAO in metabolic diseases.

Collectively, TMAO is one of the most extensively characterized gut microbiome-dependent uremic toxins across the CKM spectrum. Clinical cohorts and meta-analyzes consistently associate elevated TMAO levels with increased cardiovascular events, mortality, renal dysfunction, and features of metabolic syndrome, while mechanistic studies support direct roles in vascular inflammation, platelet hyperreactivity, hypertension, renal injury, and impaired glucose homeostasis. Although some debate remains overwhether TMAO acts as a causal mediator or a biomarker of underlying metabolic dysfunction, converging microbiome, dietary, and interventional evidence suggests that TMAO represents a key integrative link between gut dysbiosis, declining kidney function, and cardiometabolic risk.

## Indoxyl sulfate (IS)

Dietary tryptophan is an essential aromatic amino acid critical for protein biosynthesis and a biochemical precursor to metabolites that play a vital role in mammalian physiology.^79^ Tryptophan metabolism follows three major pathways in the gastrointestinal tract: (1) the direct metabolism and transformation of tryptophan into several molecules by the gut microbiota, such as indole,^80^ (2) the kynurenine pathway in host immune and epithelial cells via indoleamine 2,3-dioxygenase (IDO),^81^ and (3) the serotonin production pathway in enterochromaffin cells via tryptophan hydroxylase 1 (TPH1).^82^ These three major pathways have been reviewed elsewhere, both in health and disease.^83-85^ The gut microbiota exerts direct or indirect control over tryptophan metabolism, steering it toward microbial indole or host kynurenine and serotonin pathways.^83-85^ Here, we focus on the well-characterized gut-derived uremic toxins IS, which arises from microbial indole production from tryptophan to indole by the enzyme tryptophanase (TnaA),^86^ present in >85 Gram-negative and Gram-positive species such as *Clostridium* and *Bacteroides* species, *Escherichia coli*,^87^ followed by host hepatic oxidation and sulfation, with downstream effects in CVD, CKD and metabolic syndrome.

### Indoxyl sulfate in cardiovascular disease

IS plays a central pathogenic role in linking CKD to CVD.^38^ ^,^ ^88^ In a prospective study of patients with moderate-to-advanced CKD (*n* = 139), baseline IS levels showed an inverse relationship with renal function, and a direct relationship with aortic calcification and pulse wave velocity.^89^ In the same study, IS was identified as a strong predictor of overall and cardiovascular mortality, even after adjustment for age, gender, diabetes, albumin, hemoglobin, phosphate, and aortic calcification.^89^ IS links CKD to CVD through three major mechanisms that impair the vasculature: (1) amplifying inflammatory and oxidative stress responses, (2) promoting vascular calcification and remodeling, and (3) disrupting vascular integrity.^88^ ^,^ ^90^ ^,^ ^91^ For example, treating human monocyte-derived macrophages with IS (50-1,000 µM) demonstrated that IS functioned as a ligand for aryl hydrocarbon receptor (AhR), activating its nuclear translocation.^92^ This trigger promoted NF-κB signaling, leading to increased TNF-*α* mRNA expression and protein levels in macrophages, thereby contributing to chronic inflammation associated with CKD.^92^ Additionally, in human umbilical vein endothelial cells (HUVECs), IS treatment (25-250 µg/mL) increased ROS production and NAD(*P*)H oxidase activity while reducing nitric oxide availability.^93^ These changes were associated with endothelial dysfunction, suggesting that IS promotes a pro-atherogenic and pro-inflammatory vascular environment.^93^

Another in vitro study further demonstrated that IS activates the AhR-NF-κB signaling pathway, linking inflammation and aortic valve calcification.^94^ Primary human valvular interstitial cells (hVICs), isolated from patients with calcific aortic stenosis after valve replacement surgery, were treated with clinically relevant concentrations of IS (148 and 934 µM) under osteogenic conditions.^94^ By activating the AhR-NF-κB signaling pathway, IS induced IL-6 transcription and secretion, which subsequently promoted osteogenic differentiation marked by upregulation of the osteogenic markers BMP2 and RUNX2. These changes reflect a shift toward calcium deposition and progressive calcification, contributing to aortic valve stiffening.^94^ Furthermore, IS compromised vascular integrity in bovine pulmonary artery endothelial cells by inducing stress-mediated disruption of endothelial junctions, including VE-cadherin, *β*-catenin and p120-catenin, consequently, increasing endothelial barrier permeability.^95^ In cultured human valve endothelial cells (100 or 250 µM), IS not only enhanced calcification but also induced endothelial-to-mesenchymal transition, characterized by loss of endothelial markers (VE-cadherin, CD31 and eNOS) and increased expression of mesenchymal markers (*α*-SMA, vimentin and fibronectin).^96^

A prospective study of 165 patients hospitalized for chronic heart failure reported that individuals with higher plasma IS levels experienced a greater incidence of cardiovascular events.^97^ Kaplan–Meier analyzes further showed that elevated plasma IS concentrations (≥0.79 µg/mL) predicted the occurrence of cardiovascular events in patients with chronic heart failure.^97^ Consistently, another prospective observational study of 147 patients with CKD reported that higher serum IS levels were associated with an increased incidence of MACEs, including cardiovascular death, myocardial infarction, stroke and heart failure.^98^ Nevertheless, a meta-analysis of prospective studies involving 1,572 CKD patients demonstrated that elevated free IS levels were independently associated with increased all-cause mortality in CKD, whereas no significant association with cardiovascular events was observed,^34^ highlighting a discrepancy between mechanistic evidence and clinical outcomes. Of note, most IS studies have focused on CVD preconditioning in patients with CKD. Whether IS leads to CVD without CKD and at what stage IS exerts its pathogenesis remains unclear.

### Indoxyl sulfate in chronic kidney disease

Elevated serum IS concentrations were associated with higher inflammatory marker levels in a cross-sectional study of 149 patients with stage 3-4 CKD.^99^ In a prospective observational study of 268 patients with pre-dialysis (stages 1-5) CKD for 21 ± 3 months, serum IS levels were associated with worsening renal disease.^100^ In an observational study that enrolled 70 patients with stage 3-5 CKD and followed for 36 months, serum IS was associated with both CVD and renal disease progression to requiring dialysis.^101^ In an observational study that enrolled patients with CKD stage 2-5D and followed for 605 ± 217 days, those in the highest tertile for serum IS levels had increased cardiovascular and overall mortality.^89^ In a comparison of 24 individuals with end-stage renal disease (ESRD) and 12 control participants with normal renal function, those with ESRD exhibited a gut microbiota enriched in taxa harboring genes encoding tryptophanase.^35^ This enzyme converts tryptophan to indole, suggesting that rising serum IS levels may be partly attributable to an increased abundance of gut bacteria capable of producing these precursor metabolites.^102^ However, impaired kidney function also plays a significant role in the accumulation of IS in the plasma.^103^ Evidence from animal models has demonstrated that IS treatment exacerbates glomerulosclerosis^104^ and increases proteinuria,^105^ supporting its causative role in the progression of kidney disease.

Mechanistic *in vitro* experiments using HUVECs have demonstrated that IS stimulates ROS generation,^106^ ^,^ ^107^ which subsequently contributes to both endoplasmic reticulum stress^108^ and oxidative stress.^108^ ^,^ ^109^ These cellular stress responses activate the NF-κB signaling pathway, leading to upregulation of profibrotic gene expression.^110^ IS functions as a ligand for the AhR,^111^ which is expressed in mouse podocytes.^105^ ^,^ ^112^ When immortalized mouse podocytes are exposed to IS *in vitro*, there is an increase in pro-inflammatory gene expression with a concomitant reduction in cell viability.^105^ *In vitro*, IS triggers epithelial-to-mesenchymal transition (EMT) in immortalized renal proximal tubular cells from humans (HK-2), rats (RK-52E) and mice (PKSV-PR),^113–115^ and this EMT-inducing effect of IS has also been confirmed *in vivo* using the Dahl salt-sensitive hypertensive rat model (sex not reported).^114^ Studies conducted *in vitro* with HK2 human renal proximal tubular cells and *in vivo* in male mice subjected to half-nephrectomy have shown that IS impairs mitochondrial function.^116^

### Indoxyl sulfate in metabolic syndrome

A low-grade inflammation or “metainflammation” has been extensively linked to disturbances in glucometabolic pathways as observed in patients with obesity and T2D.^117^ The gut microbiota has emerged as a contributor to chronic low-grade inflammation that is characteristic of obesity and T2D.^117^ While the role of inflammation in insulin resistance and *β*-cell dysfunction is well studied, the upstream triggers remain less clear. The gut microbiota is a promising candidate because its metabolites interact with the immune system and metabolic pathways.^117^ Individuals with obesity have a pro-inflammatory gut microbiota, which in turn releases uremic toxins, consequently, causing inflammation and oxidative stress.^117^ For instance, circulating total IS contributed to central obesity in male patients with stable coronary artery disease (CAD, *n* = 373), partially mediated by high-sensitivity C-reactive protein, an inflammatory marker.^118^ In addition, plasma IS levels were associated with arterial stiffness in 209 patients with T2D.^119^ Urinary IS levels were associated with urinary levels of oxidative stress in patients with T2D (*n* = 255).^120^ A potential mechanism could involve breakdown of the gut epithelial barrier, leading to activation of inflammatory pathways.^75^

Taken together, the evidence shows IS as a microbiome-derived uremic toxin with strong mechanistic and clinical relevance to the cardio-renal axis of CKM syndrome. Elevated IS levels consistently predict adverse cardiovascular and renal outcomes in CKD populations and are mechanistically linked to vascular dysfunction, inflammation, oxidative stress, and progressive renal fibrosis through AhR- and NF-κB–mediated pathways. Emerging evidence further implicates IS in metabolic dysfunction, particularly in the context of chronic inflammation and insulin resistance. While most clinical data derive from CKD cohorts, the combined experimental and observational findings support IS as a pathogenic mediator rather than a passive marker of renal impairment.

## *p*-Cresol sulfate and associated metabolites

The gut microbiota is central to amino acid metabolism. The anaerobic gut microbiota utilizes tyrosine and ferments it into *p*-Cresol, releasing it as a microbial by-product.^121^ ^,^ ^122^ The biosynthesis of *p*-Cresol from dietary L-tyrosine occurs via a distinct two-step microbial pathway. Initially, L-tyrosine undergoes transamination—primarily mediated by enzymes such as tyrosine aminotransferase (TyrB)—to generate 4-hydroxyphenylpyruvate, which is subsequently converted into 4-hydroxyphenylacetate. Following this, the oxygen-sensitive glycyl radical enzyme complex *p*-hydroxyphenylacetate decarboxylase (HpdBCA), first purified from *Clostridium difficile*, catalyzes the terminal decarboxylation of 4-hydroxyphenylacetate to directly form *p*-Cresol.^123^ *In vitro* screening of 153 strains determining that *Clostridium difficile* was not only one of the top *p*-Cresol producers but it also had the highest percentage of identity of *p*-Cresol-converting enzymes mentioned above than the other strains.^124^

*p*-Cresol is a well-characterized uremic toxin that undergoes phase II metabolism, mainly in the liver and colonic mucosa, where it is transformed into water-soluble metabolites that are excreted from the body.^122^ ^,^ ^125^ Phase II metabolism includes glucuronidation and sulfation, which result in the formation of PCG or PCS, respectively.^122^ These metabolites are normally excreted via the kidneys; however, when renal function is impaired, their clearance is reduced, thereby altering gut microbiome composition and contributing to metabolic imbalance.^126^ ^,^ ^127^ Others argue that an imbalanced gut microbiome, accompanied by metabolic disturbances, may itself drive the renal pathogenesis.^127^ At present, this relationship remains a “chicken-and-egg” problem, with each component influencing the others and perpetuating a self-reinforcing cycle of dysregulation. To illustrate, *Oscillibacter* was found positively associated with inadequate fiber intake^128^ and inversely associated with the mRNA expression of zonula occludens-1 (Zo1), an essential protein for gut integrity.^129^ In addition, this genus was positively associated with four circulating uremic metabolites, including PCS, PCG, TMAO and IS, and it was correlated with accelerated kidney function decline in patients with moderate-to-severe CKD.^128^ ^,^ ^130^ Therefore, elevated levels of *p*-Cresol and its derivatives, and other uremic toxins, further amplify systemic toxicity, particularly in individuals with CKD.

### *p*-Cresol sulfate and associated metabolites in cardiovascular disease

A meta-analysis of prospective studies indicated elevated levels of PCS were associated with an increased risk of cardiovascular events among patients with chronic renal failure (*n* = 1,572 CKD patients).^34^ In addition, among the 105 patients with hypertension, those with peripheral arterial disease (*n* = 24) had higher serum PCS levels and elevated C-reactive protein (a protein marker for vascular inflammation).^131^ Elevated PCS remained an independent predictor of peripheral arterial disease, with an odds ratio of 1.154, in hypertensive patients.^131^ Furthermore, in patients with the early stage of renal failure (*n* = 202), higher total serum PCS levels were detected in the presence of CAD and were correlated with the severity of the disease.^132^ Thus, PCS has been proposed as an early biomarker for cardiovascular risk stratification, including peripheral and CAD, even before the onset of clinically apparent CVD.^132^

Mechanistically, experimental evidence indicates that PCS (and IS) contribute to the loss of cell-cell junctions by increasing tight-junction permeability *in vitro*, including HUVECs,^133^ ^,^ ^134^ and bovine aortic endothelial cells.^135^ They also induced the formation of endothelial microparticles and modulated the expression of microRNAs that regulate cellular processes.^136^ ^,^ ^137^ Additionally, in hemodialysis patients (*n* = 100), PCS levels were independently associated with endothelial microparticle count, suggesting that chronic PCS exposure may drive subtle, ongoing endothelial dysfunction, a key contributor to atherosclerosis and cardiovascular risk.^138^

Not surprisingly, *p*-Cresol, the precursor of PCS, also has detrimental effects on the cardiovascular system. It negatively impacts several key components of the system, including promoting arterial medial calcification,^91^ ^,^ ^139^ compromising the survival and barrier integrity of endothelial cells,^140-145^ and inhibiting platelet activation and aggregation *in vitro* using human and rabbit platelet-rich plasma, and further validated in mouse *ex vivo* platelet aggregation.^146^ A recent study reported a pro-thrombotic phenotype in germ-free mice colonized with bacteria engineered to produce *p*-Cresol, and showed that PCS enhanced thrombosis development.^147^ The latest evidence has been reviewed elsewhere.^148^

Another metabolite derived from *p*-cresol, though less extensively studied, is PCG. Early evidence demonstrated that both free and total PCG levels were associated with overall and cardiovascular mortality in 139 patients with CKD.^149^ More recently, a cohort study (US *n* = 4,000; EU *n* = 833) identified PCG, alongside PCS, as a gut microbiota-derived metabolite linked to MACE and mortality, independent of traditional risk factors.^150^ Of note, elevated PCG and PCS concentrations have been observed in conditions of low dietary fiber intake in both preclinical (sham and angiotensin II male C57BL/6J mice) and human studies (discovery *n* = 69; validation *n* = 1,536), highlighting that a lack of dietary fiber intake might lead to a more vulnerable gut microbiome, increasing PCG and PCS production.^128^ Remarkably, Mendelian Randomization analysis also showed that PCG, but not PCS, was causally associated with increased systolic BP in humans.^128^ PCG has attracted attention as a gut-microbiome-derived metabolite, particularly amid growing interest in prebiotic and postbiotic mechanisms. However, its specific contribution to CVD remains incompletely understood and warrants further investigation.

### *p*-Cresol sulfate and associated metabolites in chronic kidney disease

Several studies have demonstrated that plasma levels of PCS independently predict CVD and mortality in patients with CKD. In a prospective study of 175 hemodialysis patients that had a median follow-up period of 34 months, free *p*-cresol was associated with increased overall^151^ and cardiovascular^152^ mortality. These findings were concordant with a study of 112 elderly (mean age 72.6 ± 6.3 y) hemodialysis patients, who reported serum-free PCS was associated with all-cause and cardiovascular mortality during the 33.2-month follow-up period.^153^ During a 779 ± 185 day follow-up period of 139 patients (CKD stages 2–5D), serum free PCS was associated with overall and cardiovascular mortality, independent of age, vascular calcification, anemia, and inflammation.^154^ Similarly, an observational study of 499 pre-dialysis (stages 1-5) CKD patients, serum *p*-cresol concentrations predicted cardiovascular events during the 32.6 ± 2.9 month follow-up period, independently of kidney function and traditional risk scores such as the Framingham score.^155^ Additionally, urinary PCS levels have been reported as independent predictors of cardiovascular events in a prospective study of 200 patients with mild to moderate CKD (stage 1-5 CKD) during the 52-month follow-up period.^156^ In a cross-sectional study of 209 patients with diabetic nephropathy who underwent a coronary angiogram, serum PCS levels were associated with the degree of CAD, which remained significant after correction for age, BMI, gender, renal function, blood pressure, lipid profile, fasting glucose, and smoking status.^157^ In a nested case-control metabolomics study that compared 40 patients with T2D who progressed to ESRD and 40 patients with T2D who did not progress to ESRD over an 8-12 year follow-up period, those who progressed to ESRD had higher baseline plasma PCS levels compared to those who did not, while no such association was found for IS.^158^

Like IS, PCS is transported from the blood into renal tubular cells via the organic anion transporter (OAT) 1 and 3.^37^ ^,^ ^159^ Mechanistically, PCS disrupts mitochondrial function in immortalized human renal tubular cells (HK2),^116^ and has been shown to upregulate the expression of inflammatory genes^160^ and promote EMT^115^ in immortalized murine renal proximal tubular cells (PKSV-PRs). In a human proximal tubular cell line (HK-2), PCS exerts both pro-apoptotic and pro-inflammatory effects.^161^ In healthy female Wistar rats, continuous exposure to PCS at uremic concentrations increased leukocyte rolling on the peritoneal vascular endothelium, indicating heightened inflammatory activity^162^ and contributed to oxidative stress across various cell types, including leukocytes,^163^ cardiomyocytes,^164^ endothelial cells, and vascular smooth muscle cells.^165^ ^,^ ^166^ In male Sprague-Dawley rats that underwent a 5/6 nephrectomy, enhanced NAD(*P*)H oxidase activity was identified as a key driver of PCS-induced oxidative stress, which, in turn, stimulates inflammatory cytokine production and contributes to renal fibrosis.^167^ Moreover, targeted knockdown of Nox4, a specific isoform of NAD(*P*)H oxidase, reduces PCS-induced ROS generation and MCP-1 expression *in vitro* in HUVECs.^166^ Taken together, both mechanistic studies and clinical evidence consistently support a pathogenic role for PCS in the progression of renal disease, highlighting its contribution to inflammation, oxidative stress, and fibrosis in CKD.

Similarly, PCG accumulates progressively in humans as renal function declines, with both its total and free fractions increasing with decreasing eGFR, reflecting the progression of CKD.^149^ ^,^ ^168^ Elevated serum PCG concentrations were detected in uremic CKD patients; however, PCS generally reaches substantially higher total levels, with a 4-fold difference reported between these two conjugates.^169^ Higher PCG levels were independently associated with all-cause and cardiovascular mortality in two separate CKD studies (*n* = 139 and *n* = 488 CKD patients).^149^ ^,^ ^168^ Mechanistic studies showed PCG altered proximal tubule cell phenotype *in vitro*, promoting EMT in human proximal tubular cells and interacting with renal efflux transporters, such as MRP4 and BCRP.^170^ ^,^ ^171^ These interactions suggest that PCG reduced tubular excretory capacity, thereby exerting tubular toxicity that may contribute to CKD progression.

### *p*-Cresol sulfate and associated metabolites in metabolic syndrome

A cross-sectional study involving 373 CAD patients reported that higher PCS levels were associated with greater central adiposity, as indicated by the conicity index (CI) and a body shape index (ABSI), in male individuals (*n* = 257).^118^ This association was partly mediated by high-sensitivity C-reactive protein, suggesting an inflammatory pathway connecting PCS and obesity. However, no association was observed among females (*n* = 116), suggesting sex differences.^118^ Healthy mice treated with PCS (10 mg/kg, twice a day for 4 weeks) developed disrupted insulin signaling similar to clinical features typically observed in CKD.^172^ Complementary *in vitro* experiments in C2C12 myotubes revealed that PCS directly induced insulin resistance by ERK1/2-mediated impairment.^172^ Exposure to PCS reduced insulin-stimulated phosphorylation of Akt and the PI3K pathway, which is critical for glucose uptake and metabolic regulation,^172^ suggesting that PCS may directly impair tissue insulin responsiveness and resistance in CKD patients. In addition, PCS treatment (212 µM, mimicking the levels detected in ESRD) increased ROS production in mouse 3T3-L1 adipocytes *in vitro*.^173^ This oxidative imbalance can impair insulin sensitivity and trigger inflammation, linking PCS to obesity, insulin resistance, and potential cardio-renal complications.^173^ Combined with prior evidence of disrupted insulin signaling and lipid redistribution, PCS emerges as a key driver of CKD-related metabolic dysfunction.

However, surprisingly, a study population of 137 participants reported that serum *p*-cresol levels were inversely correlated with T2D.^174^ Experimental evidence showed that chronic low-dose *p*-cresol administration in male diabetic models reduced adiposity and glucose intolerance, while simultaneously enhancing insulin secretion and promoting *β*-cell proliferation.^174^ Further evidence indicated that low-dose *p*-cresol augmented glucose-stimulated insulin secretion in mouse isolated pancreatic islets, and improved glycaemic control and decreased body weight in male diabetic rats.^175^ In contrast, another study reported that *p*-cresol inhibited *Gcg* (proglucagon) expression and reduced glucagon-like peptide-1 (GLP-1) secretion in GLUTag enteroendocrine cells.^176^ Additionally, *p*-cresol supplementation in drinking water for 2 weeks suppressed transcript levels of gut hormones and modulated small intestinal transit in healthy male mice fed a Western-style diet.^176^ Collectively, these findings suggest that, at low concentrations, *p*-cresol may exert beneficial effects on glucose homeostasis and insulin secretion, whereas elevated levels are associated with gut hormone dysregulation and CKD-related metabolic complications.

Unlike PCS, PCG was reported not to impair insulin sensitivity *in vivo* or *in vitro*, indicating that PCG is relatively inert with respect to glucose metabolism and insulin resistance.^170^ Because research has primarily focused on PCS, PCG has received comparatively little attention, and its physiological role remains poorly understood. However, emerging evidence challenges the assumption that glucuronide conjugates are biologically inactive. For example, a recent study demonstrated that PCG promoted blood–brain barrier integrity, highlighting its potential systemic signaling activity and suggesting relevance for metabolic disease pathways.^177^

Overall, *p*-Cresol-derived metabolites, particularly PCS, emerge as robust predictors of cardiovascular events, mortality, renal disease progression, and metabolic dysfunction in CKD and cardiometabolic populations. Clinical and experimental studies consistently demonstrate that PCS exacerbates endothelial dysfunction, inflammation, oxidative stress, mitochondrial impairment, and insulin resistance, reinforcing its role as a pathogenic contributor within CKM syndrome. In contrast, PCG appears to exert more nuanced and context-dependent effects, with emerging evidence suggesting biological activity as a risk factor for high BP, but comparatively weaker links to metabolic dysfunction. These findings highlight the complexity of host conjugation processes and underscore the importance of considering both microbial production and host handling of cresol-derived metabolites.

## Phenylacetylglutamine (PAGln)

PAGln is a meta-organismal metabolite produced through the combined metabolic activities of the gut microbiome and the host (e.g., similar to metabolites such as TMAO, and PCS/PCG). PAGln originates from microbial metabolism of unabsorbed dietary phenylalanine that reaches the large intestine. The gut microbiota metabolize dietary L-phenylalanine into the intermediate phenylpyruvic acid (PPY) and subsequently into phenylacetic acid (PAA) (Figure 1).^178^ ^,^ ^179^ Gut microbes facilitate the initial deamination of phenylalanine through the coordinated action of microbial deaminase enzymes, such as phenylalanine dehydrogenase, tyrosine aminotransferase, and aromatic amino acid aminotransferases.^180-182^ Various gut microbes harbor these specialized enzymes; key contributors to intestinal PAA production include prominent anaerobic families such as Christensenellaceae, Ruminococcaceae, and Lachnospiraceae, as well as specific high-capacity metabolizing species including *Clostridium sporogenes, Proteus mirabilis*, and *Klebsiella pneumoniae*.^183^ This diversity illustrates the functional redundancy of gut microbes in PAA production, ensuring that the overall PAA-generating capacity remains stable even when particular microbial populations fluctuate.^179^ PAA, produced and released by the gut microbes, is absorbed into the intestinal lumen and transported via the portal circulation to the liver.^179^ In primates, PAA is mainly conjugated with glutamine to form PAGln, whereas in rodents, it is primarily conjugated with glycine to form phenylacetylglycine (PAGly).^178^ PAGln is a urine and blood metabolite present in healthy individuals, and is predominantly excreted from the body through active tubular secretion in the kidneys.^178^ ^,^ ^183-185^

### PAGln in cardiovascular disease

PAGln was firstly discovered to be associated with CVD and MACE incidence, including myocardial infarction, stroke, or death, in a discovery cohort (*n* = 1,162) and further confirmed in an independent validated cohort (*n* = 4,000).^178^ PAGln was shown to enhance platelet responsiveness in human whole blood *in vitro* and accelerate platelet clot formation in the FeCl3-induced carotid artery injury thrombosis model.^178^ In the same study, PAGln was identified as interacting with G protein-coupled adrenergic receptors, including α2A, α2B, and β2 adrenergic receptors, triggering downstream cellular responses, and with *β*-blocker carvedilol (1.5 g/kg for one week) attenuated PAGln-induced heightened thrombosis risk.^178^ This discovery catalyzed the research of PAGln in CVD. Several clinical studies have suggested that the elevated levels of PAGln are associated with the pathology of cardiovascular diseases, including heart failure, coronary heart disease, atherosclerosis, and cardiac arrhythmia.^186^ For instance, plasma PAGln levels were found to be dose-dependently associated with heart failure and indices of severity independent of traditional risk factors and renal function in two large clinical cohorts (discovery *n* = 3,256; validation *n* = 829).^187^ In the same study, it was revealed that both PAGln and its rodent equivalent, PAGly, directly contribute to heart failure-related changes.^187^ These include reduced sarcomere contraction in cardiomyocytes and decreased expression of natriuretic peptide B gene in cultured cardiomyoblasts and murine atrial tissue.^187^

Remarkably, PAGln accelerates cellular senescence. In two healthy cohorts (discovery *n* = 132, validation *n* = 80), increases in PAGln levels and microbial gene clusters that promote its biosynthesis weredetected in older individuals.^188^ In the same study, both cellular models (HUVECs and IMR-90, primary fetal lung fibroblasts) and experimental intervention in healthy male mice (daily intraperitoneal 50 mg/kg PAGln injection for 4 weeks) showed that PAGln induced mitochondrial dysfunction and promoted ROS accumulation, leading to DNA damage and activation of senescence pathways, thereby contributing to ageing.^188^ This is particularly relevant because endothelial senescence is a key driver of age-related CVD. Consistent with these findings, elevated levels of PAA and its derivative PAGln were associated with ageing in both humans (*n* = 7,303) and aged mouse models (>24 months).^189^ Ageing increased PAA-producing microbial pathways, especially in *Clostridium sp.* ASF356. Colonization of young mice with this bacterium increased circulating PAA and induced endothelial senescence through overstimulating mitochondrial hydrogen peroxide (H2O2) production, thereby exacerbating the senescence-associated secretory phenotype.^189^

Furthermore, higher plasma PAGln levels were correlated with a greater risk of coronary heart disease in a large prospective cohort of 1,520 females.^190^ In another independent study, PAGln predicted MACE in more than 1,500 high-risk hypertensive patients over a 3 year period.^191^ It appeared that PAGln might have a sex-different effect in CVD, as using PAGln as a MACE predictor was more accurate in females, particularly in patients with systolic blood pressure above 130 mmHg and those taking angiotensin-converting enzyme inhibitors (ACEIs).^191^ Nevertheless, further studies are needed, and PAGln should be considered as a potential biomarker for risk stratification to improve CVD prognosis.

### PAGln in chronic kidney disease

Phenylethylamine (PEA) is predominantly oxidized into PAA,^192^ a compound found at concentrations of 3.49 ± 0.33 mmol/L in 41 patients with ESRD.^193^ In contrast, PAA levels in 39 healthy controls were below 10 μmol/L (the limit of detection), representing a greater than 300‑fold increase in concentration, the highest reported for any uremic toxin.^193^ ^,^ ^194^ Mechanistically, cell culture studies have demonstrated that PAA increases ROS production in primary vascular smooth muscle cells from Wistar–Kyoto rats,^195^ disrupts mitochondrial function in human conditionally immortalized renal proximal tubule epithelial cells (ciPTEC),^196^ and directly inhibits renal tubular efflux mechanisms in human embryonic kidney cells (HEK293).^197^ The gut microbiota plays a significant role in PAGln production, as shown by a 14-fold increase in serum PAGln levels that were observed in a comparative study of 9 dialysis patients with an intact colon compared with 6 dialysis patients who had undergone colectomy.^198^ Additionally, PAGln concentrations have been linked to the relative abundance of bacterial families within the Clostridiales order.^199^ In a study comparing 25 hemodialysis patients and 16 healthy control subjects, the total serum concentration of PAGln was found to be over 100 times higher in the dialysis group (5.2 ± 2.5 mg/dl) than in the normal subjects (0.05 ± 0.02 mg/dl).^200^ These levels were inversely associated with eGFR in healthy populations (*n* = 4,439),^201^ positively associated with the progression to ESRD in patients with diabetes (*n* = 80) in a case-control study,^158^ and linked to increased cardiovascular risk in both pre-dialysis (*n* = 488)^199^ and dialysis-dependent (*n* = 394)^202^ CKD patients.

### PAGln in metabolic syndrome

Elevated plasma PAGln was reported in stroke patients with T2D compared to those without T2D in two separate clinical cohorts (combined patients with T2D *n* = 50; without T2D *n* = 69). ^203^ When transplanting fecal microbiota from stroke patients with T2D to male Sprague-Dawley rats, these animals exhibited a more severe brain injury and higher circulating levels of PAGln compared to those rats that received FMT from stroke patients without T2D.^203^ Male rats receiving intraperitoneal injections of PAGln (50 mg/kg, 3 doses administered hourly, followed by the middle cerebral artery occlusion procedure after 30 min of the final injection) had increased systemic inflammatory cytokine TNF-*α*, oxidative stress damage and neutrophil extracellular traps formation in brain tissues, leading to more severe brain infarction in stroke.^203^ Another study reported that elevated plasma PAGln levels were strongly associated with poor wound healing outcomes in T2D patients (discovery *n* = 85; validation *n* = 115).^204^ When translated to an *in vivo* setting, with PAGln intervention (daily intraperitoneal injection or continuous mini-pump infusion) exhibited persistent inflammatory signaling, delayed wound closure and neovascularisation and chronic inflammation in both diabetic and non-diabetic mice.^204^ Nevertheless, the study did not report any examination of sex differences, even though both sexes were included in both clinical and animal studies. On the other hand, surprisingly, urinary PAGln levels were inversely associated with BMI in two independent epidemiological studies (discovery *n* = 1,880; validation *n* = 444).^205^ While another study found no evidence that obesity was associated with PAGln production.^187^ These limited and controversial findings reflect that the knowledge about PAGln in metabolic syndrome is still in its early stages and remains to be elucidated.

In summary, PAGln represents a recently identified gut microbiome-derived metabolite with growing relevance to cardiovascular and renal pathology. Strong clinical associations link PAGln to MACE, heart failure severity, thrombosis risk, and mortality, supported by mechanistic studies demonstrating adrenergic receptor-mediated platelet hyperreactivity and cellular senescence. In CKD, PAGln accumulates markedly as renal function declines and predicts adverse outcomes, whereas evidence linking PAGln to metabolic dysfunction remains limited and conflicting. Together, these findings position PAGln as a promising biomarker and potential mediator of cardiovascular risk within the gut-kidney-heart axis, warranting further mechanistic and interventional investigation.

## Imidazole propionate

ImP is a microbial metabolite produced by the gut microbiota from histidine. Histidine is converted by the bacterial enzyme histidine ammonia-lyase (*hutH*) to the intermediate urocanate, which is subsequently converted into ImP via the enzyme urocanate reductase (*urdA)*.^206^ Key producers of ImP include *Streptococcus mutans* and *Eggerthella lenta.* ^207^ Although histidine is an essential amino acid, dietary histidine intake is not the determining factor influencing circulating ImP levels.^208^ From a dietary perspective, ImP levels have been positively correlated with saturated fat intake and negatively correlated with fiber and unsaturated fat intake.^208^ ^,^ ^209^ These findings suggest that unhealthy dietary patterns that adversely alter microbial ecology are key drivers of ImP production.

### Imidazole propionate in cardiovascular disease

In a cross-sectional study of 107 overweight and obese adults without diabetes, higher circulating levels of ImP were correlated with diastolic BP.^210^ Elevated ImP levels were observed in a cohort of patients with chronic heart failure (*n* = 166) compared with healthy controls (*n* = 69),^211^ and levels increased with declining LVEF.^212^ Furthermore, using two large, independent cohorts (*n* = 1,985 and *n* = 2,155), elevated ImP levels were associated with higher five-year mortality in heart failure patients.^212^ In patients with CAD, higher circulating ImP levels were associated with more advanced disease and predicted MACE and mortality in three independent cohorts comprising patients with acute and chronic coronary syndromes.^213^ When stratifying patients by both Imp and TMAO levels, individuals with higher levels of both metabolites had the highest risk of MACE, and both metabolites were stronger predictors of MACE at three years than traditional risk factors, including body mass index, age, and diabetes.^213^ Mechanistically, ImP appears to impair endothelial migration and repair, promote vascular inflammation through PI3K AKT FOXO1 signaling, and accelerates atherosclerosis in mice.^214^ ImP has been demonstrated to drive atherosclerosis and plaque formation via effects on the imidazoline 1 receptor.^215^ Together, epidemiological and mechanistic evidence support ImP as a microbiota‑derived metabolite linking gut microbial dysbiosis to cardiovascular disease progression and adverse outcomes.

### Imidazole propionate in chronic kidney disease

ImP levels are higher in patients with diabetic nephropathy or receiving peritoneal dialysis, compared with individuals with T2D without kidney impairment or healthy controls.^216^ ^,^ ^217^ Concentrations were positively correlated with albuminuria and inversely correlated with eGFR.^217^ In a preclinical model of diabetes, the db/db mouse, intraperitoneal injection of 100 μL ImP (20 mg/kg/day) for 4 weeks worsened albuminuria and glomerulosclerosis, suggesting that ImP itself may be nephrotoxic.^217^ Assuming a mouse weight of 45g, this would equate to a daily intraperitoneal dose of 0.9 mg/day (6.42 μmol), which, if distributed throughout the total body water of the mouse (27 mL), would equate to a concentration of 238 µM. Another preclinical study administered male C57BL/6J mice with daily intraperitoneal ImP at 100 μg (714 nmol) for 12 weeks and found increased urinary albuminuria, glomerular and tubular injury, higher serum IL-1β, and activation of ROS NLRP3 signaling, all of which were attenuated by rapamycin.^218^ Assuming a mouse weight of 30g, with total body water of 18 mL, this would equate to 39.7 µM. These calculations are provided to provide context for the supraphysiological doses utilized in these preclinical experiments in comparison with plasma concentrations in peritoneal dialysis patients which have been reported as 0.412 ± 0.121 µM,^216^ whilst ImP was reported in the nanomolar range in CAD^214^ and T2D participants.^208^ While evidence is emerging, the pathogenic role of ImP in kidney disease progression is currently less compelling than for metabolic and cardiovascular disease.

### Imidazole propionate in metabolic syndrome

ImP levels were higher in people with diabetes than in healthy controls, despite no differences in dietary histidine intake, suggesting that in the context of T2DM, there is greater microbial capacity for ImP production.^208^ This has been confirmed experimentally; when stool samples from patients with T2DM were incubated with histidine, higher levels of ImP were produced than when fecal samples from healthy control participants were incubated with histidine.^207^ Mechanistically, administration of exogenous ImP has been demonstrated to disrupt insulin signaling through the p38γ-p62-mTORC1 axis, and to blunt metformin action through inhibitory AMPK phosphorylation.^207^ ^,^ ^219^ Together, these findings support ImP as a plausible microbiota-derived mediator of metabolic dysfunction that may be contributing to CKMS.

## Uremic toxins: association versus causation

As reviewed above, several microbiome-derived uremic toxins seem to be independent predictors of cardiovascular, renal, and metabolic complications. Reduced renal function makes it difficult to disentangle whether uremic toxins themselves or the underlying renal impairment come first. As highlighted above, there is strong evidence that some of these metabolites contribute to diseases associated with CKM syndrome. However, mechanistic evidence remains uneven: some metabolites have robust mechanistic data (e.g., TMAO and IS in CVD), while others are supported only by correlative or controversial findings, or less mechanistic evidence (e.g., PCG and PAGln in metabolic syndrome). Experimental models, which are often used to determine the role of metabolites, are not always useful in the setting of uremic toxins, as under normal renal function, metabolites are rapidly cleared, making it challenging to achieve consistently high levels over prolonged periods. Few trials have selectively lowered individual uremic toxins. For example, a high-fiber/SCFA randomized, placebo-controlled, crossover, double-blind clinical trial lasting three weeks significantly reduced BP,^25^ *p*-Cresol and PCG, but not PCS, in patients untreated for hypertension.^128^ Tools such as Mendelian randomization, which identify causal relationships based on genetic variation,^220^ may be helpful in this setting—for example, while PCG is rapidly cleared by healthy kidneys, using a Mendelian randomization approach, PCG was causally associated with hypertension.^128^ Before therapies can be designed to reduce the levels of these metabolites as a new way to treat CKM syndrome and its associated diseases, a causal framework is needed. Integrating mechanistic studies, longitudinal cohorts, interventional trials, and systems biology will be essential to move the field from correlation toward causation.

## Future directions

Uremic toxins exert wide-ranging effects across the spectrum of CKM syndrome, influencing processes from insulin resistance and immune activation to renal and cardiovascular pathology. However, their role has typically been examined in isolation or primarily in patients with established renal dysfunction. With the emergence of CKM syndrome as a unified framework, there is a pressing need to better characterize and stratify patients according to CKM criteria, systematically analyze uremic toxin profiles, and develop an accessible database such as the EUtox database (https://database.uremic-toxins.org/soluteList.php). Such an approach will identify which toxins most strongly predict disease progression and outcomes at different stages and can be targeted for therapy. Importantly, sex-specific differences warrant attention, as metabolites such as TMAO and PAGln exhibit distinct patterns between males and females. However, most studies, especially in animals, still focus solely on males, making it difficult to assess conclusions and their relevance to both sexes.

**Figure 2.**
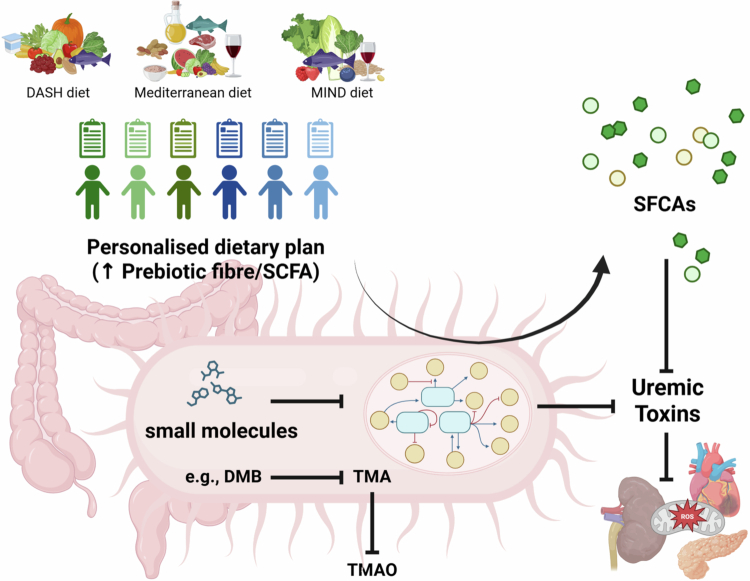
From dietary to microbiome-targeted interventions to reduce microbial uremic toxins. Traditional healthy dietary patterns that are high in dietary fiber and personalized dietary strategies can optimize substrate delivery to the gut microbiota, for example, by incorporating prebiotic fibers and/or postbiotic supplements, such as short-chain fatty acids (SCFAs), to limit toxin production. Small-molecule inhibitors can target microbial metabolic pathways to reduce or block the formation of gut-derived uremic toxins. For instance, 3,3-dimethyl-1-butanol (DMB) blocks TMA production, thereby reducing TMAO generation and circulating levels in the host. Figure created in BioRender.

Several potential interventions aim to mitigate the impact of uremic toxins (Figure 2). A key strategy involves harnessing gut microbes, which produce and release these toxins or their precursors into the host system. Targeting microbial pathways can, therefore, reduce toxin production and associated adverse effects. For example, 3,3-dimethyl-1-butanol (DMB) is a small molecule that inhibits microbial choline TMA-lyase activity, effectively lowering TMAO levels in mice fed high-choline or L-carnitine diets.^221^ Similarly, blocking CutC and CutD, the major microbial TMA-generating enzymes, reduced plasma TMAO levels by >95% for up to 3 days, and reversed diet-induced platelet aggregation and thrombosis risk in female mice with induced arterial injury.^222^ Another promising approach involves inhibiting *p*-Cresol production: a novel compound, 4-hydroxyphenylacetonitrile, reduced *p*-cresol generation by 99% in *Clostridioides difficile in vitro*.^223^ This lead compound warrants further investigation in fiber-deficient settings to determine its *in vivo* efficacy in reducing *p*-cresol and related metabolites.

Additional small-molecule inhibitors have been developed to target specific microbial functions within the human gut microbiome.^224^ Tyrosine decarboxylase inhibitors prevent the microbial conversion of tyrosine into tyramine, a metabolite that can affect blood pressure regulation.^224^ These advances underscore the growing potential of small-molecule inhibitors as a frontier in microbiome science and therapeutic development, offering both mechanistic insights and opportunities for precision interventions.

Dietary intake strongly shapes the gut microbiome; therefore, personalized and improved nutritional strategies are essential for preventing or mitigating disease development. In a highly controlled feeding trial in healthy people, red meat intake, but not white meat or non-meat protein, more than doubled both plasma and urinary TMAO, indicating strongly the role of dietary precursors in TMAO production.^225^ The current recommendations for daily fiber intake are 30 g for males and 25 g for females, which are considered sufficient to maintain gastrointestinal health and regularity.^226^ However, to reduce the risk of chronic diseases, including CVD, diabetes, and certain cancers, the optimal target is 38 g/day for males and 28 g/day for females.^226^ Indeed, an intervention with a high-resistant-starch supplement that was acetylated and butyrylated (high-amylose maize starch), which delivered an ~8-fold increase in SCFAs, significantly reduced blood pressure in 20 patients untreated for hypertension by 6.1 mmHg^25^ and reduced levels of *p*-cresol and PCG.^128^ Responders for this intervention had significantly lower levels of fiber and wholegrains at the start of the trial,^227^ supporting personalized nutritional approaches to identify the best candidates for such interventions. Resistant starch interventions have also been shown to reduce IS and PCS in patients undergoing hemodialysis^228^ and animal models.^229^ IPA in high-fiber diets, such as the Dietary Approaches to Stop Hypertension (DASH) and the Mediterranean diet, lower the incidence of CVD in both epidemiological studies and clinical trials.^230^ A meta-analysis that included 21 randomized controlled trials involving 700 CKD participants, with fiber supplementation ranging from 6–50 g/day for typically more than four weeks, showed that dietary fiber supplementation modestly but significantly reduced serum uremic toxins, including PCS, IS, and blood urea nitrogen, as well as inflammatory markers, such as IL-6 and TNF-α.^231^

Direct administration of propionate and butyrate in mice reduced renal injury markers, inflammation, and fibrosis, while improving overall kidney function.^232^ In addition, a targeted multi-biotic formulation for CKD (SynCKD), comprising the probiotic *Lactobacillus johnsonii* NCC533, a prebiotic (1% cellobiose), and a postbiotic (1% butyrate source), significantly lowered IS and PCS levels and enhanced renal outcomes in rodent models.^233^ SCFA supplementation and other postbiotic strategies have emerged as promising approaches to reduce uremic toxins and slow CKD progression.^234^ Collectively, these findings highlight the therapeutic potential of microbiome-targeted interventions as a novel avenue for reducing gut-derived toxins and their associated complications, and to mitigate CKM syndrome.

## Conclusion

Growing evidence indicates that gut-derived uremic toxins, particularly TMAO, IS and PCS, play a pivotal mechanistic role in the development and progression of CKM syndrome. These metabolites arise from alterations in gut microbial composition and function, coupled with impaired intestinal barrier integrity, and act as key mediators linking the gut microbiome to systemic cardiometabolic and renal injury. Through activation of inflammatory, oxidative, endothelial, and metabolic pathways, microbiome‑dependent toxin accumulation contributes to cardiovascular dysfunction, insulin resistance, and progressive kidney damage. As kidney function declines, their accumulation further amplifies cardiovascular risk, accelerates renal fibrosis, worsens insulin resistance, and perpetuates the gut-kidney-heart axis of injury. Collectively, these observations position the gut microbiome not only as a determinant of uremic toxin generation but also as a modifiable therapeutic target. Strategies that restore microbial balance, reduce toxin production, or personalized nutritional plans may offer new opportunities to mitigate the interconnected pathways that drive CKM syndrome.

## Abbreviations

ABSI: a body shape index
ACEIs: angiotensin-converting enzyme inhibitors
AGEs: advanced glycation end-products
AhR: aryl hydrocarbon receptor
BMP2: bone morphogenetic protein 2
BP: blood pressure
CAD: coronary artery disease
CI: conicity index
CKD: chronic kidney disease
CKM: cardiovascular-kidney-metabolic
CVD: cardiovascular disease
eGFR: estimated glomerular filtration rate
EMT: epithelial-to-mesenchymal transition
ESRD: end-stage renal disease
FLI: fatty liver index
FMO: flavin-containing monooxygenase
FMT: fecal microbiota transplant
GLP-1: glucagon-like peptide-1
HUVECs: human umbilical vein endothelial cells
hVICs: human valvular interstitial cells
IDO: indoleamine 2,3-dioxygenase
IS: indoxyl sulfate
KIM-1: kidney injury molecule-1
LPS: lipopolysaccharide
MACE: major adverse cardiovascular events
MAPK: mitogen-activated protein kinase
MASLD: metabolic dysfunction-associated steatotic liver disease
NADPH: nicotinamide adenine dinucleotide phosphate
OAT: organic anion transporter
PAA: phenylacetic acid
PAGln: phenylacetylglutamine
PAGly: phenylacetylglycine
PCG: *p*-Cresol glucuronide
PCS: *p-*cresol sulfate
PEA: Phenylethylamine
PPY: phenylpyruvic acid
ROS: reactive oxygen species
RUNX2S: Runt-Related Transcription Factor 2
SCFAs: short-chain fatty acids
STEMI: ST-segment elevation myocardial infarction
T2D: type 2 diabetes
TDO: tryptophan 2,3-dioxygenase
TMA: trimethylamine
TMAO: trimethylamine-N-oxide
TPH1: tryptophan hydroxylase 1
Zo-1: zonula occludens-1
